# Scanning laser ophthalmoscopy retroillumination: applications and illusions

**DOI:** 10.1186/s40942-022-00421-0

**Published:** 2022-09-30

**Authors:** Martin A. Mainster, Thomas Desmettre, Giuseppe Querques, Patricia L. Turner, Gerardo Ledesma-Gil

**Affiliations:** 1 Department of Ophthalmology, University of Kansas School of Medicine, Prairie Village, KS USA; 2 Centre de Retine Medicale, Marquette-Lez-Lille, France; 3 Ophthalmology Department, University Vita-Salute, IRCCS San Raffaele Scientific Institute, Milan, Italy; 4 Retina Department, Institute of Ophthalmology, Fundacion Conde de Valenciana, Chimalpopoca 14, Colonia Obrera, Cuauhtemoc, 06800 Mexico City, Mexico

**Keywords:** Confocal, Light scattering, Optical coherence tomography, Pseudocolor, pseudo3D, Retinal imaging, Retroillumination, Scanning laser ophthalmoscope, Shape-from-shading, Transillumination, Visual illusion

## Abstract

Scanning laser ophthalmoscopes (SLOs) are used widely for reflectance, fluorescence or autofluorescence photography and less commonly for retroillumination imaging. SLOs scan a visible light or near-infrared radiation laser beam across the retina, collecting light from each retinal spot as it’s illuminated. An SLO’s clinical applications, image contrast and axial resolution are largely determined by an aperture overlying its photodetector. High contrast, reflectance images are produced using small diameter, centered apertures (confocal apertures) that collect retroreflections and reject side-scattered veiling light returned from the fundus. Retroillumination images are acquired with annular on-axis or laterally-displaced off-axis apertures that capture scattered light and reject the retroreflected light used for reflectance imaging. SLO axial resolution is roughly 300 μm, comparable to macular thickness, so SLOs cannot provide the depth-resolved chorioretinal information obtainable with optical coherence tomography’s (OCT’s) 3 μm axial resolution. Retroillumination highlights and shades the boundaries of chorioretinal tissues and abnormalities, facilitating detection of small drusen, subretinal drusenoid deposits and subthreshold laser lesions. It also facilitates screening for large-area chorioretinal irregularities not readily identified with other en face retinal imaging modalities. Shaded boundaries create the perception of lesion elevation or depression, a characteristic of retroillumination but not reflectance SLO images. These illusions are not reliable representations of three-dimensional chorioretinal anatomy and they differ from objective OCT en face topography. SLO retroillumination has been a useful but not indispensable retinal imaging modality for over 30 years. Continuing investigation is needed to determine its most appropriate clinical roles in multimodal retinal imaging.

## Background

Confocal scanning laser ophthalmoscopes (SLOs) are widely used to produce conventional reflectance monochromatic or multiwavelength fundus images. Some confocal SLOs have an additional retroillumination mode that can produce “pseudo-three-dimensional” (pseudo3D) images [1–10]. In their widely-used reflectance-mode (direct-mode), SLOs record images from light reflected directly back (retroreflected) from chorioretinal structures. In their retroillumination-mode (indirect-mode), SLOs create images from light or infrared radiation that transilluminates chorioretinal structures as it returns indirectly from deeper choroidal and scleral layers.

Retroillumination highlights and shades the boundaries of chorioretinal tissues and abnormalities, facilitating detection of small drusen, subretinal drusenoid deposits and subthreshold laser lesions [1–10]. It also facilitates identification of large-area chorioretinal irregularities not readily displayed in other en face retinal imaging modalities [11–14]. Shaded boundaries create the perception of elevation or depression in imaged structures [15–22], a characteristic of retroillumination but not standard reflectance SLO imaging. This perception is an illusion rather than an objective three-dimensional representation of chorioretinal anatomy as provided by optical coherence tomography (OCT). We review and analyze the tissue-optics, clinical applications and limitations of SLO transillumination imaging.

## Confocal SLOs

Clinical confocal SLOs sweep a 10–15 μm diameter laser spot across the fundus and collect light point-by-point from sequentially illuminated retinal sites [23–26]. The retina is exposed only to one small diameter, low irradiance laser spot at a time so SLO imaging is comfortable and safe for patients and provides high lateral image resolution [23–26].

An SLO’s laser beam enters the retina roughly perpendicular to the plane of the retina [23–26]. Light absorption and light scattering including reflection and refraction attenuate the laser beam progressively as it descends to greater chorioretinal depths [27–29]. Light scattering is most prominent at tissue interfaces and surfaces where there are changes in refractive index, including the sclera, retinal pigment epithelium (RPE) and internal limiting membrane (ILM) [30, 31]. Longer wavelengths (red and near-infrared) penetrate more deeply into the choroid than shorter wavelengths (blue) largely because optical radiation absorption by the primary chorioretinal absorbers (melanin, hemoglobin and macular pigment) is lower at longer wavelengths [27, 32, 33].

**Fig. 1:**
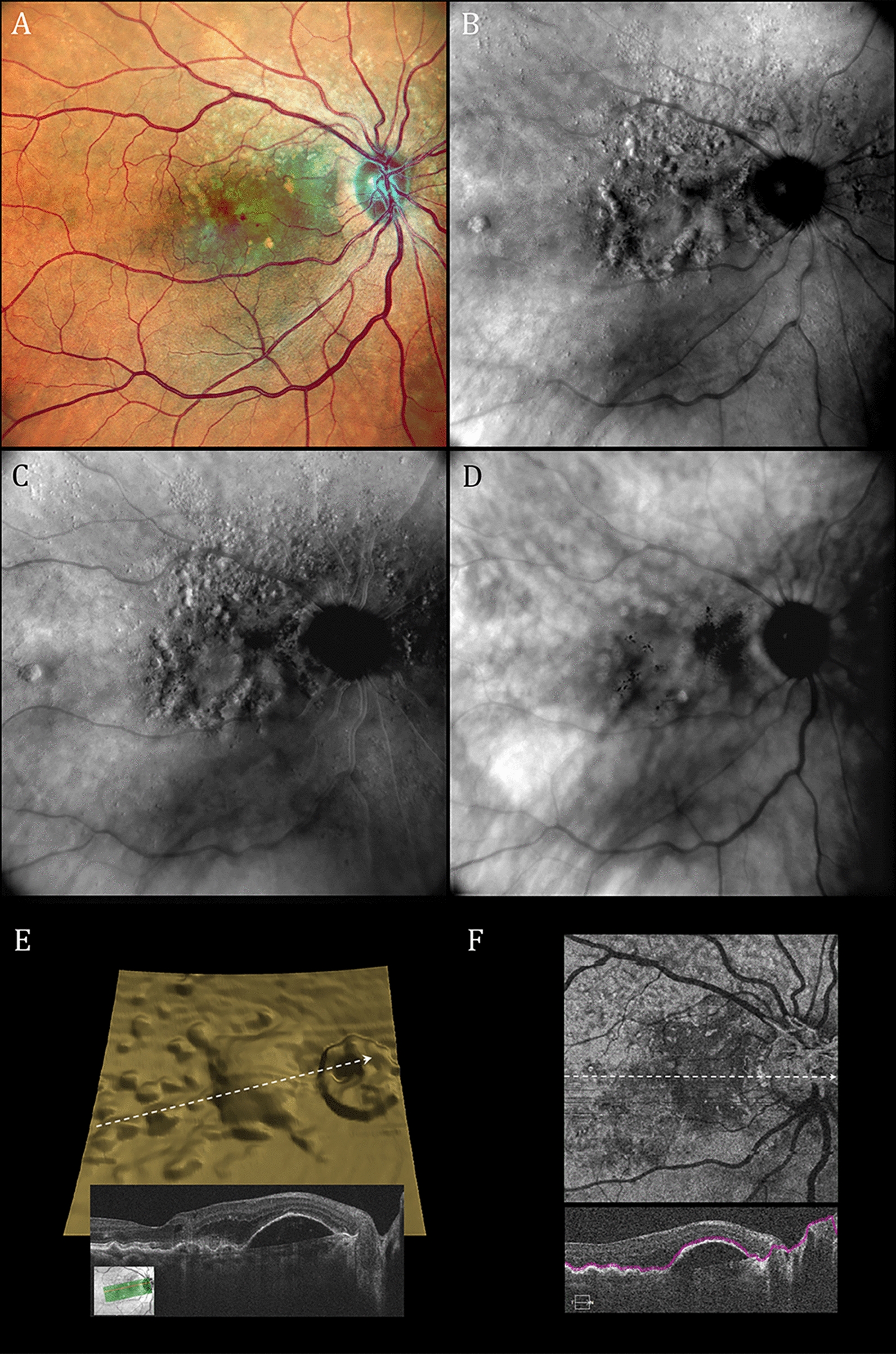
Reflectance and retroillumination scanning laser ophthalmoscope (SLO) as well as optical coherence tomography (OCT) images of the right retina of a 75-year-old female with neovascular age-related macular degeneration, small subretinal hemorrhages, a large vascularized retinal pigment epithelial detachment and numerous drusen. **A** Reflectance multiwavelength SLO image. **B** Retroillumination SLO image taken with a deviated-to-the-right (DR) confocal aperture. **C** Retroillumination SLO image taken with a deviated-to-the-left (DL) aperture. **D** Retroillumination SLO image taken with a ring aperture (RA; annular aperture). Prominent lesion highlighting and border shading in **B** and **C** are absent with the Mirante ring aperture but present in published images with annular apertures using other SLO retroillumination systems. [1–4, 49, 54] **E** Segmented retinal pigment epithelium three-dimensional OCT topography differing from retroillumination shading patterns in **B** and **C** (inset shows B-scan along white arrow). **F** En face structural OCT image corresponding roughly to tissue planes of RPE topography in **E** and drusen in **B** and **C**

**Fig. 2:**
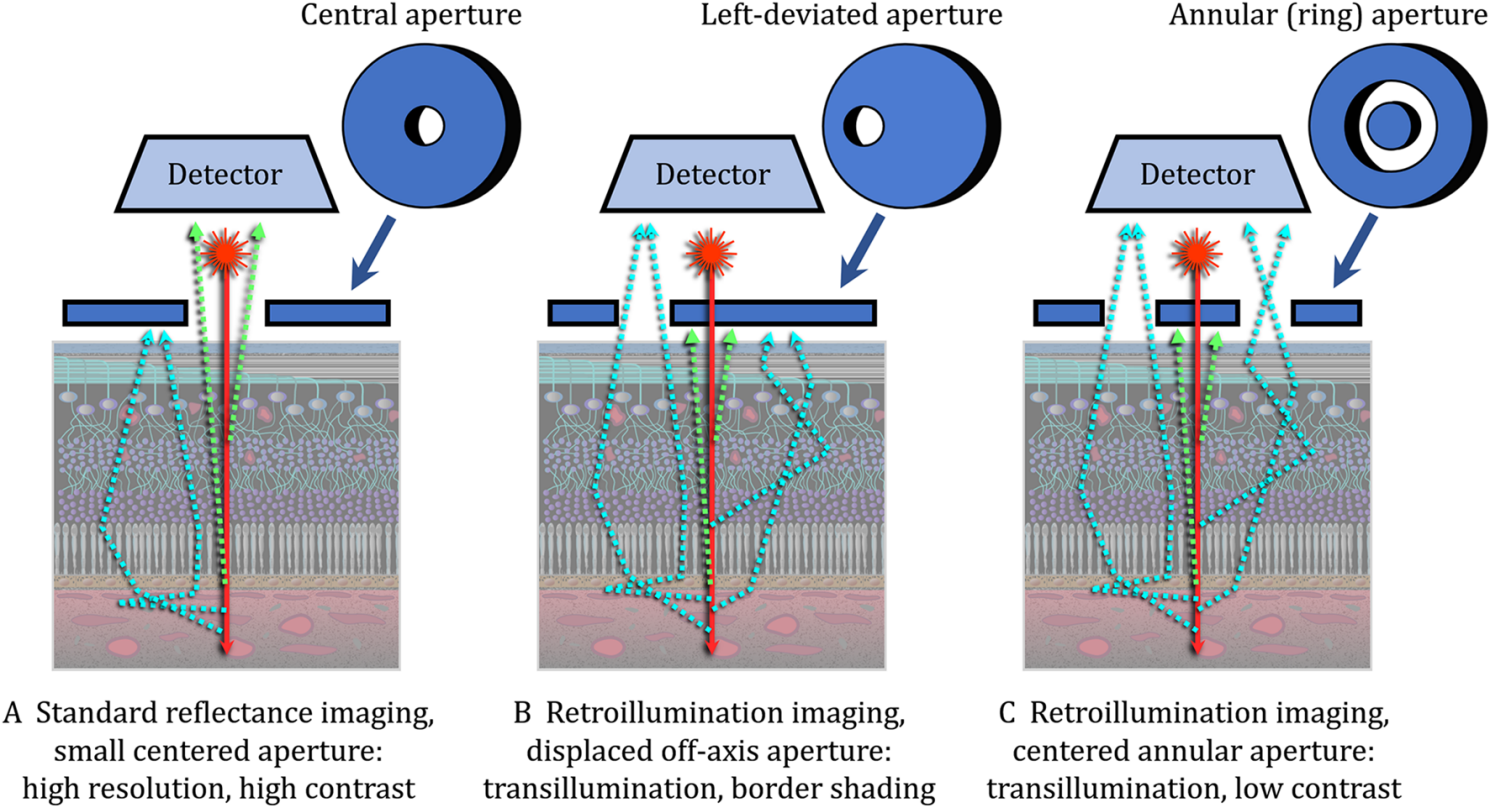
Scanning laser ophthalmoscopy (SLO) confocal apertures for reflectance (direct-mode) and retroillumination (indirect-mode) SLO imaging. **A** In standard reflectance imaging, a centered confocal aperture limits light collection only to photons reflected “directly” back to the photodetector from the illuminated retinal spot, [25] thereby increasing retinal image contrast by blocking photons from other fundus locations that could cause veiling glare at the photodetector. [25, 64] **B** and **C**. In retroillumination imaging, an aperture deviated laterally (**B**) or an annular aperture (**C**) blocks retroreflected light and collects only photons scattered “indirectly” back to the SLO’s photodetector. Laterally-deviated (**B**) or annular (**C**) apertures assure asymmetric or symmetric light collection in the retinal plane, respectively. In the Mirante system, asymmetric retroillumination imaging (**B**) transilluminates, highlights and shades borders of imaged chorioretinal structures whereas symmetric annular (ring) aperture light collection (**C**) provides low contrast transillumination images

One or more lasers are used to produce monochromatic or multicolor SLO images, respectively. Monochromatic images are used in OCT systems to localize B-scans [34]. Multicolor images are created by combining monochromatic images [35, 36]. Multicolor confocal SLOs are useful alternatives for fundus cameras despite color imaging differences [37–40]. Some confocal SLOs can produce both (1) conventional reflectance images (reflectance-mode) as shown in Fig. 1A and (2) pseudo3D retroillumination-mode images as shown in Figs. 1B, 1C and 1D. We used a Mirante SLO (Nidek Co., Ltd., Gamagori, Japan) and a Cirrus 5000 HD-OCT (Carl Zeiss AG, Jena, Germany) to acquire the SLO and OCT images in this report, respectively.

## Reflectance-mode imaging

Confocal SLOs produce reflectance images (Figs. 1A and 2A) from laser light scattered directly back from the fundus (hence the term direct-mode for reflectance imaging) [4, 25, 41]. The system is called confocal because the scanning laser beam and a small aperture centered in front of the SLO’s photodetector are both focused on the same retinal location [25]. The small confocal aperture (1) restricts light collection to photons retroreflected from the illuminated retinal focal point, (2) blocks veiling glare that could be produced by photons scattered back to the photodetector from other retinal locations and (3) narrows depth of focus (increases axial resolution) [1, 25, 29, 42]. Reducing depth of focus (optical slab thickness) also eliminates eyelash artifacts encountered with larger aperture ultra-widefield SLO systems [43].

Confocal apertures in SLO systems provide axial resolutions of only ~ 300 μm, [34, 44–47] equivalent to total macular thickness (285 μm) [48]. Thus, even tomographic SLO systems could not offer the intraretinal detail available with OCT’s 3 μm axial resolution [49, 50]. Larger area apertures used for retroillumination-mode or ultra-widefield SLO imaging have even lower axial resolutions (larger optical slab thickness) [1, 25, 42].

## Retroillumination-mode imaging

Confocal SLOs produce pseudo3D images by replacing the small confocal aperture centered in front of the SLO’s photodetector (Fig. 2A, reflectance-mode) with a laterally-displaced (deviated) aperture (Fig. 2B) or a centered annular (ring-shaped) aperture (Fig. 2C) [1, 4, 5, 7, 25, 51]. Laterally-displaced and annular apertures both (1) collect initially scattered light that returns indirectly to the detector (hence the term indirect-mode for retroillumination imaging) and (2) reject light retroreflected directly back from the laser-illuminated retinal spot. In essence, retroillumination-mode imaging uses light that is rejected in reflectance-mode imaging and reflectance-mode imaging uses light rejected in retroillumination-mode imaging. Retroillumination images are created from light transilluminating chorioretinal structures (retroillumination) rather than reflected off their inner surfaces [29, 44]. The Mirante SLO is the only currently-available commercial SLO system that provides retromillumination-mode imaging. Past SLO transillumination studies have used earlier versions of this system, research SLOs and a commercial device that is no longer available [1–6, 25]. Retroillumination-mode Mirante imaging uses infrared laser radiation (790 nm) because it penetrates more deeply into the choroid than visible (400 – 700 nm) light [7, 27, 28, 32]. Chorioretinal structures are transilluminated by multiply-scattered photons returning from the sclera and deep choroidal layers. Structures are rendered visible by reflection and refraction, much in the same way that an empty, transparent wine glass is visualized [25, 29]. Tissue irregularities and abnormalities are visible as lesions or retinal areas shaded laterally from a light to a dark border. Tissue shading facilitates rapid detection of abnormalities that are more difficult to detect with other en face multimodal imaging methodologies. Figures 1B and 1C are typical Mirante pseudo3D images taken with deviated-to-the-right (DR) and a deviated-to-the-left (DL) apertures, respectively. Figure 1D was taken during the same imaging session using the Mirante annular ring aperture (RA) that produces low contrast images without the tissue shading needed for pseudo3D effects.

## Pseudo3D imaging

Lesion shading and pseudo3D effects are often ascribed to the asymmetric off-axis (deviated right or left) positioning of the proprietary retro-mode apertures of the Mirante and its predecessor F-10 SLO system [5–7, 9, 52, 53]. Indeed, Mirante’s symmetric ring-aperture typically produces only low-contrast, non-pseudo3D images. Nonetheless, symmetric annular (ring) apertures in other commercial and experimental SLO systems create pseudo3D images with non-horizontal as well as horizontal shading gradients [1–4, 49, 54]. Contrast generated in retroillumination-mode imaging depends on aperture design, which the Mirante’s manufacturer does not disclose. In general, smaller area apertures produce higher image contrast and narrower depth of focus [25, 55, 56].

SLO retroillumination image shading has been compared to lunar mountain shadowing [7, 9]. For example, it was reported that “In retromode imaging, the lateral incident light generates a shadow on the side opposite to the incident light source, resulting in an appearance of the retina that shows some resemblance to the lunar landscape” [9]. That comparison is problematic because an SLO’s laser beam is incident perpendicular not oblique to the retinal surface so there is no “side opposite to the incident light source.” Moreover, the analogy is misleading clinically because accurate height information can be extracted from lunar shadows but not from SLO retroillumination image border shading. Specifically, photogrammetry can determine building, tree and impact crater-wall height from solar shadows but that is not possible with retromode boundary shading because it is caused by multiple intraretinal light scattering events.

## Pseudo3D is shape-from-shading

The shape-from-shading percept causes the depth perceived in pseudo3D retroillumination-mode SLO images [57]. Shading and border structure help an observer identify a three-dimensional object’s shape but shape-from-shading percepts can produce false illusions of depth in two-dimensional objects [15, 16, 22]. Visual perception integrates sensory data with sensory biases acquired from prior experiences (“priors”), including the expectation that the shading of natural objects is caused by a single light source [15–22]. Shape-from-shading pseudo3D illusions have also been observed previously in scanning electron microscopy [58] and retinal differential interference contrast microscopy images. [59, 60]

**Fig. 3:**
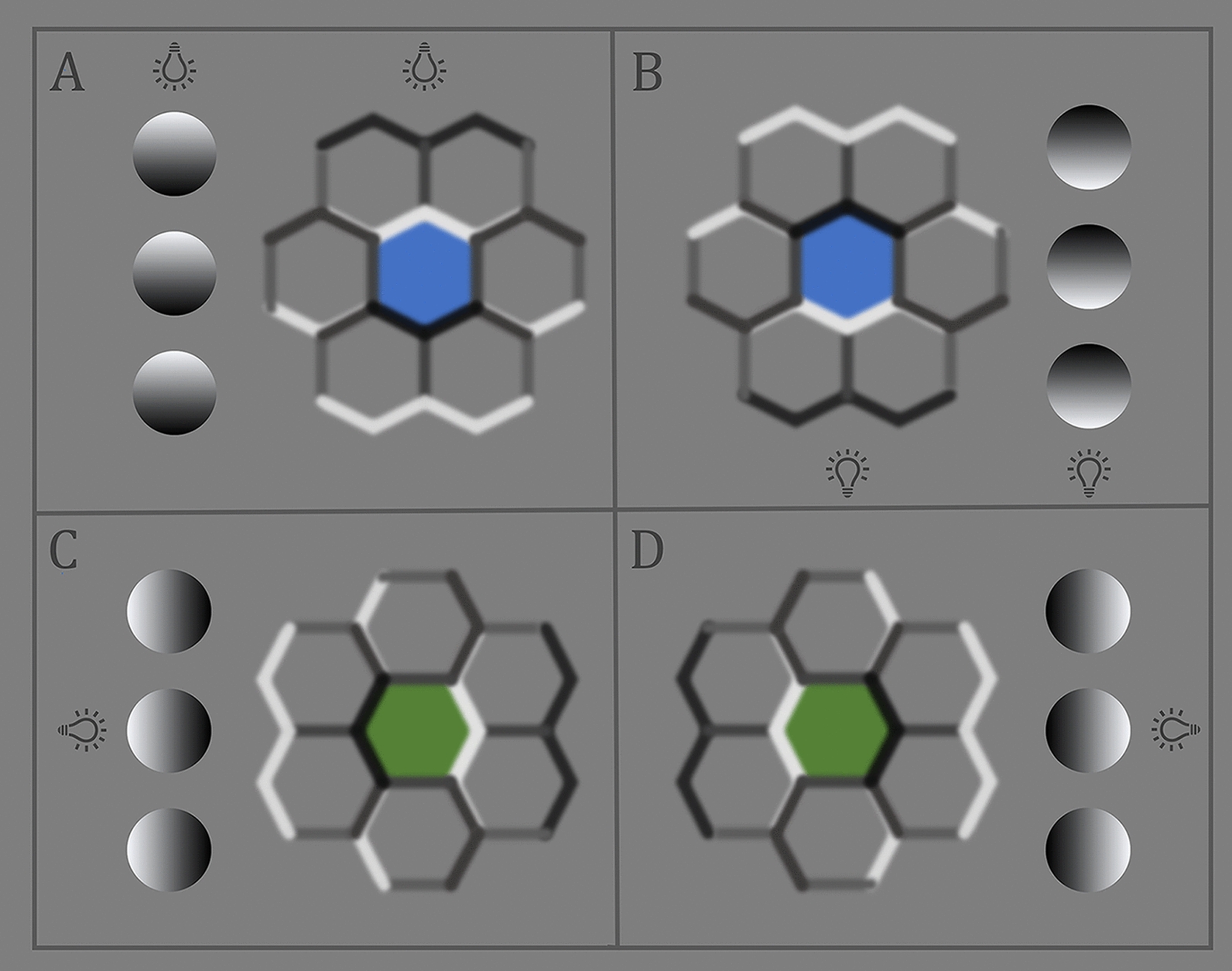
The visual system infers shape-from-shading of two-dimensional hexagonal [18] or circular [15] objects largely biased on the assumption that they are three dimensional objects illuminated by a single light source. There is no objective three-dimensional depth information in any of these two-dimensional patterns which are perceived to be elevated or depressed depending on whether the observer believes that the light source illuminating them is located superior or inferior (**A** and **B**), or to the left or right (**C** and **D**) of the image. The depth perceived in them is a visual illusion. Elevation and indentation can be reversed by rotating any of these objects 180 degrees in the horizontal plane or by an observer consciously changing the location of the light source believed to be responsible for the boundary shading

Figure 3 demonstrates how the visual system uses shape-from-shading to perceive elevation and depth in intrinsically two-dimensional images. Perceived elevation or indentation depends on an observer’s belief that a light source is located superior, inferior or to the left or right of these hexagonal [18] or circular [15] patterns. There is no objective three-dimensional depth information encoded in Fig. 3’s patterns, as is present in a stereophotograph or hologram. Perceived depth in Fig. 3 is just a visual illusion. Apparent elevation or indentation can be reversed merely by rotating these objects 180 degrees in the horizonal plane or by an observer consciously changing the location of the light source believed to be causing the image shading.

## SLO pseudo3D and OCT

**Fig. 4:**
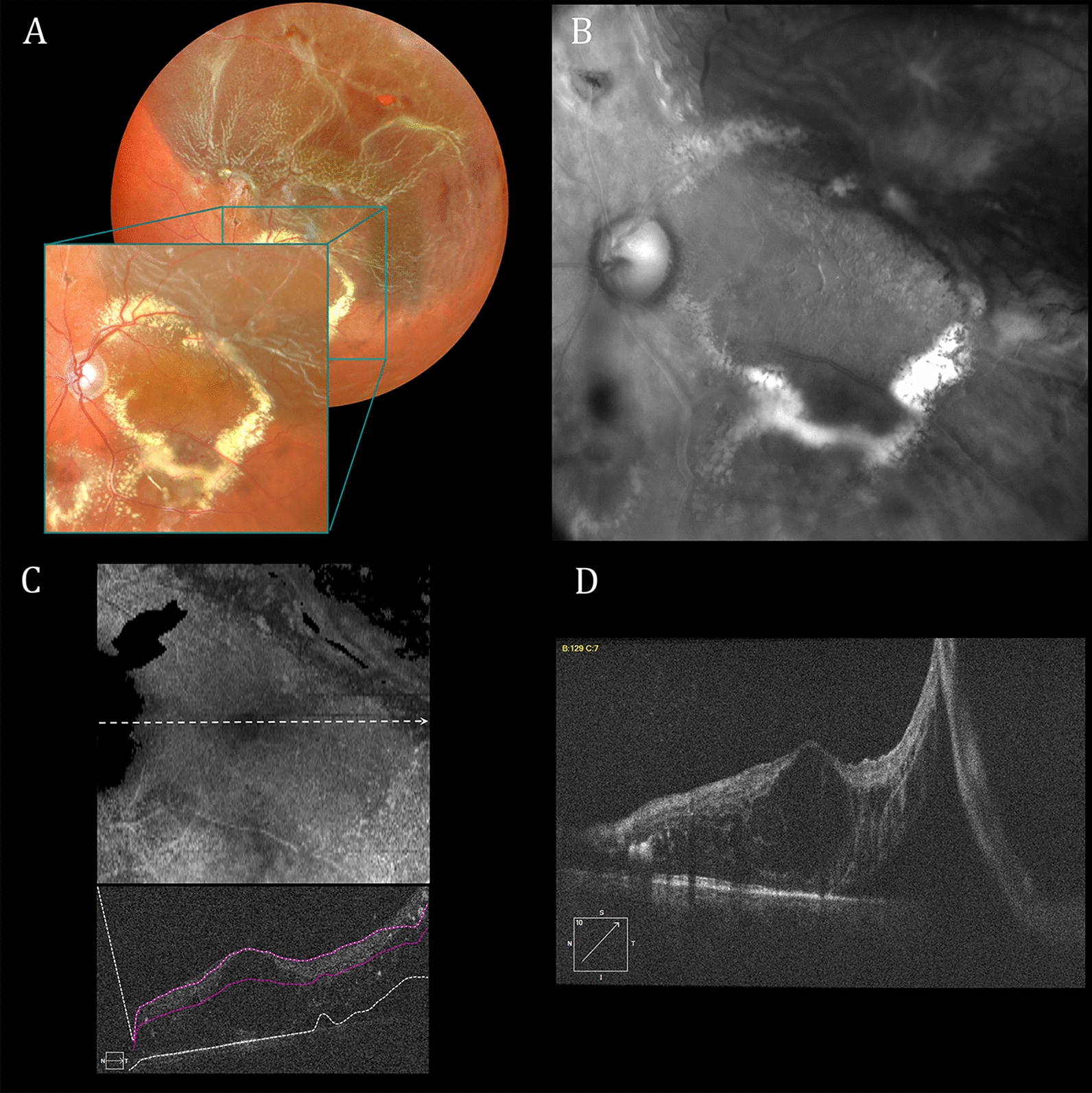
Reflectance and retroillumination scanning laser ophthalmoscope (SLO) as well as optical coherence tomography (OCT) images of the left retina of a 46-year-old man with Coats disease treated multiple times over a 15-year period with laser photocoagulation and intravitreal anti-VEGF therapy. **A** Reflectance multiwavelength SLO imaging shows a rhegmatogenous retinal detachment that extends centrally from a retinal hole in the superior-temporal mid-periphery. **B** Retroillumination SLO imaging with a deviated-to-the-left (DL) aperture shows widespread posterior pole retinal crinkling and cystoid changes associated with the macula-off detachment. **C** En face and associated B-scan OCT images display central wrinkling less prominently than in **B**. **D** OCT B-scan documenting a macula-off retinal detachment and prominent associated cystoid abnormalities

**Fig. 5:**
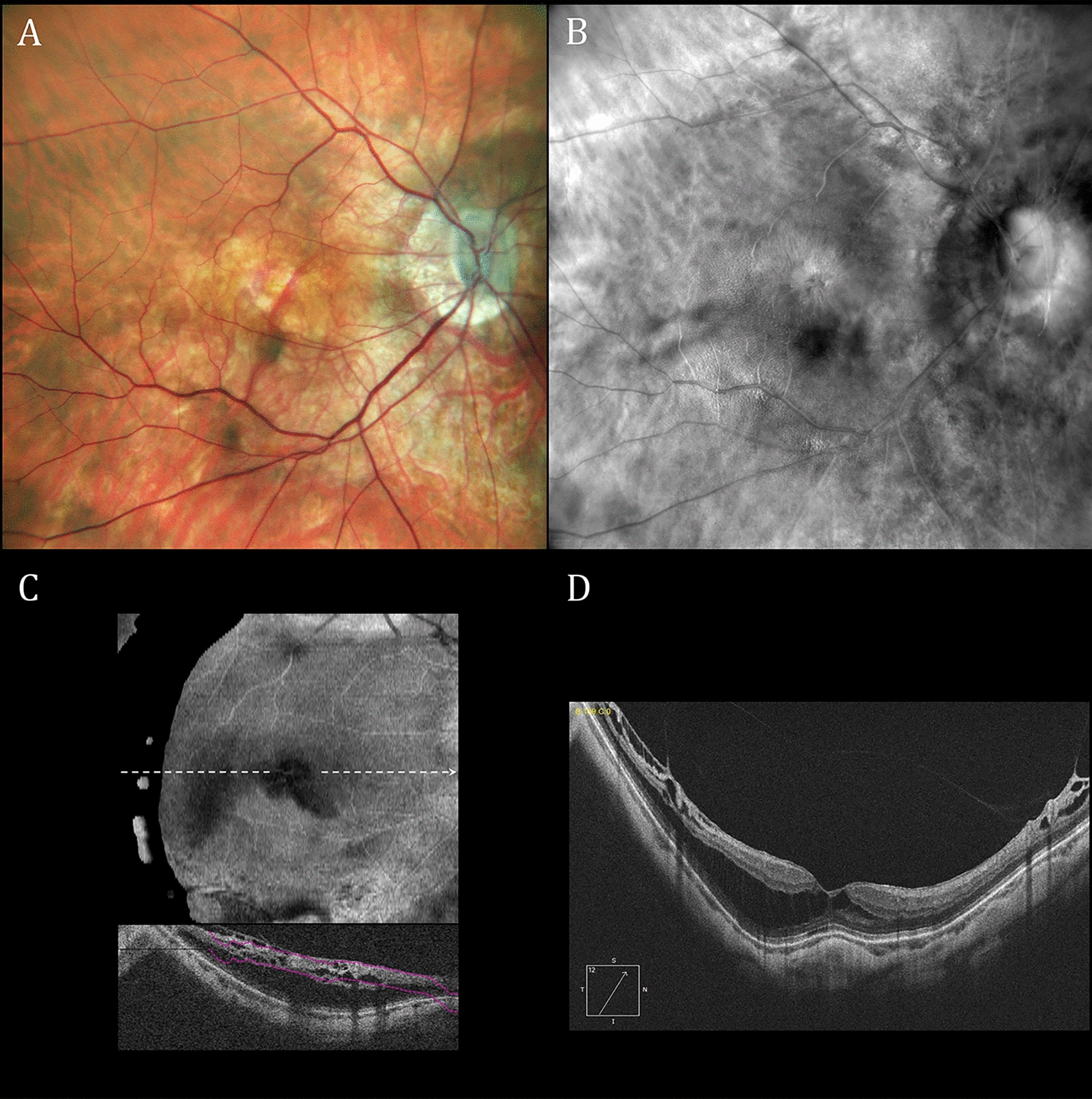
Reflectance and retroillumination scanning laser ophthalmoscope (SLO) as well as optical coherence tomography (OCT) images of the right retina of a 67-year-old male with myopic macular retinoschisis. **A** Reflectance multiwavelength SLO image shows a prominent myopic scleral crescent and reduced fundus pigmentation. **B** Retroillumination SLO image taken with a deviated-to-the-right (DR) aperture shows widespread posterior pole wrinkling due to macular retinoschisis. **C** En face and associated B-scan OCT images show less detailed and widespread cystic changes than in **B**. **D** OCT B-scan documents central and temporal macular retinoschisis

The exaggerated topographic irregularities perceived in Fig. 1B and C are illusory percepts that differ from OCT segmentation topography (Fig. 1E) and en face imaging (Fig. 1F) taken during the same clinical visit. En face OCT allows users to select the depth and thickness of an imaged retinal slab. Conversely, retroillumination-mode SLO’s full-thickness macula slab [44, 45, 47, 48] highlights only prominent chorioretinal disturbances, as shown in Figs. 1, 4 and 5. Border shading and accenting can identify abnormalities not readily visualized with other en face imaging modalities but they cannot provide OCT’s quantitative estimates of chorioretinal depth. Once identified, however, SLO retroillumination irregularities are readily evaluated with other forms of multimodal imaging.

Tomography and stereoscopy have been used in the past to obtain chorioretinal depth information from confocal SLO technology. (1) Scanning laser tomography developed for optic disc topography analysis was used for volumetric macular edema [34, 61, 62]. (2) Paired reflectance SLO images taken at different angles were viewed stereoscopically to identify elevation in fluorescein angiographic lesion [63]. Neither method approaches OCT’s precision and clinical utility. Sequential confocal SLO and OCT imaging facilitates proper clinical interpretation of retroillumination SLO images.

## Conclusions

Highlighting and shading of chorioretinal structure boundaries in SLO retroillumination images is useful for detecting small drusen, subretinal drusenoid deposits and subthreshold laser lesions. Additionally, it provides useful images of large-area chorioretinal irregularities not readily apparent with other en face retinal imaging modalities. Retroillumination SLO images are strikingly different from those produced by other en face imaging modalities. Their pseudo3D depth illusion caused by the shape-from-shading percept is perhaps their most distinguishing characteristic. It facilitates chorioretinal lesion screening and identification but does not provide objective depth information. Retroillumination-mode SLO imaging has been a useful but not indispensable retinal imaging modality for over 30 years. Continuing investigation is needed to determine its most appropriate clinical roles in multimodal retinal imaging.

## Abbreviations

ILM: Internal limiting membrane
OCT: Optical coherence tomography
RPE: Etinal pigment epithelium
SLO: Scanning laser ophthalmoscope
DR: SLO deviated-to-the-right aperture
DL: SLO deviated-to-the-left aperture
RA: SLO ring aperture
